# Modulation of sensorimotor cortex by repetitive peripheral magnetic stimulation

**DOI:** 10.3389/fnhum.2015.00407

**Published:** 2015-07-14

**Authors:** Eugen Gallasch, Monica Christova, Alexander Kunz, Dietmar Rafolt, Stefan Golaszewski

**Affiliations:** ^1^Department of Physiology, Medical University of GrazGraz, Austria; ^2^Institute of Physiotherapy, University of Applied Sciences FH-JoanneumGraz, Austria; ^3^Department of Neurology, Paracelsus Medical University of SalzburgSalzburg, Austria; ^4^Center for Medical Physics and Biomedical Engineering, Medical University of ViennaVienna, Austria

**Keywords:** afferent-induced facilitation, cortical plasticity, motor cortex, peripheral magnetic stimulation, TMS, fMRI

## Abstract

This study examines with transcranial magnetic stimulation (TMS) and with functional magnetic resonance imaging (fMRI) whether 20 min of repetitive peripheral magnetic stimulation (rPMS) has a facilitating effect on associated motor controlling regions. Trains of rPMS with a stimulus intensity of 150% of the motor threshold (MT) were applied over right hand flexor muscles of healthy volunteers. First, with TMS, 10 vs. 25 Hz rPMS was examined and compared to a control group. Single and paired pulse motor evoked potentials (MEPs) from flexor carpi radialis (FCR) and extensor carpi radialis (ECR) muscles were recorded at baseline (T0), post rPMS (T1), 30 min post (T2), 1 h post (T3) and 2 h post rPMS (T4). Then, with fMRI, 25 Hz rPMS was compared to sham stimulation by utilizing a finger tapping activation paradigm. Changes in bloodoxygen level dependent (BOLD) contrast were examined at baseline (PRE), post rPMS (POST1) and 1 h post rPMS (POST2). With TMS facilitation was observed in the target muscle (FCR) following 25 Hz rPMS: MEP recruitment curves (RCs) were increased at T1, T2 and T3, and intracortical facilitation (ICF) was increased at T1 and T2. No effects were observed following 10 Hz rPMS. With fMRI the BOLD contrast at the left sensorimotor area was increased at POST1. Compared to inductions protocols based on transcutaneous electrical stimulation and mechanical stimulation, the rPMS induced effects appeared shorter lasting.

## Introduction

It is well known that a period of afferent stimulation is able to facilitate corticomotor excitability, and to induce outlasting neuromodulatory effects within the sensorimotor cortex. Such effects had been shown following trancutaneous electrical stimulation (TES), at stimulation intensities either above motor threshold (MT; Ridding et al., 2000), below MT but above sensory level (Hamdy et al., 1998), or even at stimulation intensities below sensory level (Golaszewski et al., 2004), see Chipchase et al. (2011) for review. Further such effects were demonstrated with mechanical stimulation, either applied to tendons (Forner-Cordero et al., 2008), to muscles (Rosenkranz and Rothwell, 2003; Marconi et al., 2008) or to the whole hand (Christova et al., 2011). In this context also repetitive peripheral magnetic stimulation (rPMS) was probed in order to induce supraspinal neuromodulatory effects. However with rPMS the focus was on therapeutic interventions, in attempt for treatment of upper-limb disabilities (Nielsen et al., 1996; Struppler et al., 2003; Krause and Straube, 2005; Flamand et al., 2012; Krewer et al., 2014), and sensory deficits (Heldmann et al., 2000; Kerkhoff, 2003).

In the present study, we examined whether rPMS promotes modulatory effects within the sensorimotor motor cortex of normal subjects. Concerning the activation of peripheral nerves, magnetic simulation underlies similar ionic mechanisms as TES (Nilsson et al., 1992). However if compared to TES, the magnetic field induced eddy currents penetrate into deeper tissue regions (Barker, 1999), thus activating fast conducting proprioceptive and somatic nerve structures (Behrens et al., 2011). To this muscle contractions induced by rPMS are recognized as less painful (Bischoff et al., 1994), supporting evidence that axons from superficial pain receptors became less excited (Struppler et al., 1996). Furthermore, if compared to TES, magnetic stimulation is less able to elicit maximal responses with shortest conduction times in median and ulnar nerves (Olney et al., 1990), and therefore is not suited for the neurography of hand and fingers (Bischoff et al., 1995). Nevertheless, concerning the induction of cortical plasticity, comparable effects are expected with rPMS.

For the assessment of afferent-induced neuromodulatory effects non-invasive techniques such as transcranial magnetic stimulation (TMS) and functional magnetic resonance imaging (fMRI) showed adequate. Based on single and paired pulse TMS protocols the recorded motor evoked potentials (MEP) contain information about corticospinal exitability (Burke et al., 1993) and intracortical inhibition/disinhibition (Kujirai et al., 1993). MEPs are commonly recorded over relaxed muscles, therefore with TMS neuroplastic changes in the resting motor cortex are revealed. On the contrary with fMRI, the estimated blood oxygenation level dependent (BOLD) contrast reveals neuroplastic changes in the activated cortex (Wu et al., 2005). Motor tests, for instance finger tapping induce activation within a wide sensorimotor network (Moriyama et al., 1998) thus enabling evaluation of afferent-induced effects beyond the motor cortex (Christova et al., 2013).

The strength of the afferent-induced effects depends on stimulation parameters like frequency, waveform, amplitude (Chipchase et al., 2011), but also on the period and number of applications (Marconi et al., 2008). With a single period of stimulation application post-effects up to 2 h were found (Charlton et al., 2003) indicating long term potentiation (LTP) like mechanisms in the genesis of cortical plasticity (Kaelin-Lang et al., 2002). For the current study we examined whether a period of 20 min rPMS applied to the forearm muscles induces post stimulatory effects in the corresponding area of the motor cortex. The induced effects were evaluated with TMS and fMRI. With TMS excitability changes following induction protocols with rates of 10 and 25 Hz were tested and compared to a 25 Hz sham stimulation protocol over calf muscles. Due to resonance-like phenomena in the somatosensory system (Snyder, 1992) a rate of 25 Hz is expected to evoke stronger neuromodulatory effects. With fMRI cortical activations following the 25 Hz induction protocol were tested by utilizing a finger tapping paradigm.

## Materials and Methods

### TMS Experiments

#### Subjects and Study Design

**Figure 1:**
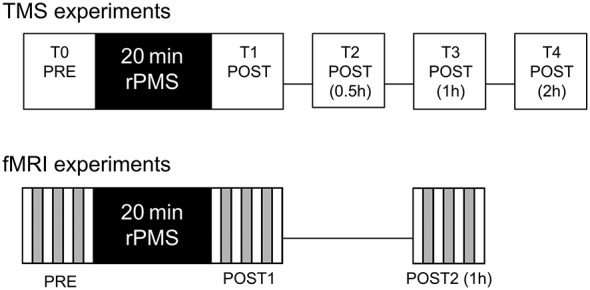
**Experimental procedures for the transcranial magnetic stimulation (TMS) and functional magnetic resonance imaging (fMRI) assessments.** TMS assessments were carried out before (T0), immediately after (T1), 0.5 h after (T2), 1 h after (T3) and 2 h after (T4) the repetitive peripheral magnetic stimulation (rPMS) treatment. fMRI assessments were carried out before (PRE), immediately after (POST1) and 1 h after (POST2) the rPMS or sham treatment.

Twelve healthy right handed subjects (3 ♂ and 9 ♀, mean age 27.75 ± 2.45) were recruited for the TMS experiments. Three rPMS induction protocols were tested in a within-subjects design: 10 Hz rPMS over hand flexor muscles (HAND10), 25 Hz rPMS over hand flexor muscles (HAND25), and for control 25 Hz rPMS over calf muscles (LEG25). These protocols were delivered in a random order at three different days. An inter-session interval of at least 5 days was provided in order to avoid any lasting post-stimulation effects. A complete experimental session (upper, Figure 1) included five TMS assessments and one intervention in the following order: TMS before rPMS (T0), rPMS intervention for 20 min, TMS post rPMS (T1), post 0.5 h (T2), post 1 h (T3), and TMS post 2 h (T4). The study was approved by the local Ethics Committee.

#### Application of rPMS

Repetitive peripheral magnetic stimulation was delivered using the Power MAG 100 clinical stimulator.^1^ To achieve predominantly focal activations in nerve terminals supporting wrist and finger flexors, a figure of eight coil (Type PMD70) was used for the stimulations. The stimulation was delivered via preprogramed pulse trains: 10 s on and 10 s off over 20 min. Thus the total number of applied single impulses was 6000 at 10 Hz, and 15,000 at 25 Hz, but the number of pulse train induced hand contractions was equal for both stimulation protocols. For the 25 Hz pulse trains two coils were alternated in order to avoid heating up from intermitted current flow.

For hand stimulation rPMS was applied to the volar site of the right forearm to the area of finger and wrist flexor muscles. Subjects were seated on an armchair, the right forearm was placed on a pillow next to the body in supinated position and the elbow was slightly flexed (130°). For leg stimulation subjects were seated in the same position but the right leg was lifted on a step while rPMS was delivered to the calf muscles. The coil was positioned so that the handle is perpendicular to the arm (leg) and was kept in that position from the investigator. Before application of rPMS the individual MT was determined via single pulse trials. MT of peripheral magnetic stimulation was defined as the lowest stimulation intensity, which was able to induce a visible contraction in the hand and finger flexors, or respectively a visible twitch in the calf muscles. The mean MT (expressed as percentage of maximum stimulator output) was 17.00 ± 1.48% for HAND10, 17.00 ± 1.41% for HAND25 and 17.88 ± 0.83% for LEG25. Then the stimulation intensity was increased 50% above MT in order to induce motor contractions. Thus the applied mean stimulation intensity was 25.67 ± 2.19% for HAND10, 25.58 ± 1.98% for HAND25, and 27.00 ± 1.07% for LEG25.

#### TMS Assessments and EMG Recordings

TMS assessments were performed with two Magstim 200 stimulators and a Bistim module (The Magstim Company, Whitland, Dyfed, UK), connected to a double 70 mm coil (Type P/N9925-00). The coil was placed over the left motor cortex at the optimal site for stimulating the contralateral right flexor carpi radialis (FCR) and extensor carpi radialis (ECR) muscles. The optimal coil position was marked on an electroencephalogram (EEG) cap and fixed via an adjustable stand. MEPs were recorded using surface Ag-AgCl electrodes positioned in a bipolar montage (interelectrode distance, 2 cm) on the skin overlying FCR and ECR muscles. EMG signals were amplified and filtered (band pass 8–2000 Hz), digitized with sampling rate of 10 kHz, and stored on disc (DasyLab 8.0 software package) for offline analysis.

The TMS procedure included assessment of resting MT, MEP recruitment curve (RC), intracortical facilitation (ICF) and short-latency intracortical inhibition (SICI). MT was defined as the lowest stimulus intensity at which five out of ten consecutive TMS applications leaded to a MEP of at least 0.05 mV on the relaxed FCR and ECR. MEP RCs were recorded at stimulation intensities 100, 110, 120, 130, 140, 150 and 160% of MT and were administered in a random order (Christova et al., 2011). SICI and ICF were assessed using the paired-pulse paradigm (Kujirai et al., 1993) with interstimulus intervals (ISI) of 3 and 13 ms. The conditioning and test stimuli were 80 and 120% of the corresponding MT at the actual assessment (at time levels T0, T1, T2, T3 and T4).

#### Data Analysis and Statistics

For MT, presented as fraction of maximum stimulator intensity (% mean ± SEM), a two-factorial ANOVA (analysis of variance) was carried out with within-subject factors *time* (5 levels: T0, T1, T2, T3, T4) and *rPMS* (3 levels: HAND10, HAND25, LEG25). When a significant interaction with *rPMS* was found in the two-factorial ANOVA, one-way ANOVAs were conducted with within-subject factor *time* for each stimulation protocol separately.

For the MEP RCs amplitudes were calculated as percentage of maximum mean MEP (mean ± SEM) at baseline (T0)—which was usually at 160% of MT intensity—for each subject individually. The MEP amplitudes were analyzed individually for each stimulus intensity and each muscle applying a repeated measures ANOVA with within-subject factors: *time* (5 levels: T0, T1, T2, T3, T4) and *rPMS* (3 levels: HAND10, HAND25, LEG25). In case of significant main or interaction effects, follow-up ANOVAs for each stimulation protocol and each muscle were carried out. Additionally the slope steepness of the RCs was calculated as linear regression and analyzed with factors *time* (5 levels: T0, T1, T2, T3, T4) and *rPMS* (3 levels: HAND10, HAND25, LEG25).

For paired pulse MEP (SICI and ICF) the amplitudes were calculated as fraction of the single pulse MEP for each subject (mean ± SEM). Then repeated measures ANOVAs were performed for FCR and ECR separately for SICI and ICF with within-subject factors: *time* (5 levels: T0, T1, T2, T3, T4) and *rPMS* (3 levels: HAND10, HAND25, LEG25). In case of significant main and interaction effects, follow-up ANOVAs for each rPMS level were conducted separately.

For statistical testing a significance level of 0.05 was applied. Further, where ANOVA showed significance, Bonferroni corrected pairwise *post hoc* comparisons were carried out.

### fMRI Experiments

#### Subjects and Study Design

A collective of 30 healthy right handed subjects were recruited for the fMRI experiments. The subjects were allocated randomly to a verum group (*n* = 15, 7 ♂ and 8 ♀, mean age 31.44 ± 9.76) receiving 25 Hz rPMS over right forearm muscles (STIM), and to a control group (*n* = 15, 9 ♂ and 6 ♀, mean age 36.44 ± 12.38) receiving no rPMS (NOSTIM).

The experimental protocol contained four fMRI sessions: baseline assessement (PRE), 20 min treatment period receiving STIM or NOSTIM, post-stimulation assessment after the treatment (POST1), and second post-stimulation assessment one hour after treatment (POST2), see lower Figure 1. For treatment subjects were removed from the scanner and during this period the verum group was stimulated in the same way as described above. For treatment of the control group stimulation intensity was set to a level below MT (5% of stimulator output). Also during the pause between POST1 and POST2 subjects were removed from the scanner. The activation paradigm for both groups was self-paced finger-to-thumb tapping with the right hand according to a procedure described recently (Christova et al., 2013). The total experimental time was about 100 min. The study was approved by the local Ethics Committee.

#### Scanning Procedure

A clinical scanner (Siemens Magnetom Trio Tim syngo MR B15), equipped with an EPI-capable gradient system and Siemens-issued 32-channel head coil was used to carry out the experiments. The scanning procedure was equal as recently described (Christova et al., 2013 ) and is repeated here briefly. For fMRI, we used T2*—weighted single shot echo-plantar sequences (TR 2.570 ms; FA 78°; TE 30 ms; matrix = 64 × 64; 40 slices; 3 mm slice thickness; and 0.75 mm slice gap). Scans of the whole brain with 40 slices parallel to the bicomissural plane were obtained. In every run a series of 75 sequential volume images was acquired. In addition, high-resolution anatomical images were acquired for each subject. Thus a 3D magnetization-prepared, rapid acquisition gradient echo was used with the parameters: TR = 2300 ms; TE = 2.91 ms; 160 slices; slice thickness = 1.20 m; in-plane resolution = 1.0 mm × 1.0 mm and FA = 9°.

Ten scans of rest (A) alternated with 10 scans of activation (B) were repeated three times in each run according the scheme: A B A B A B A. During the runs, participants were instructed to stay in relaxed position with hands placed on the abdomen, to keep their eyes closed throughout the experimental procedure and not to lift up the hand during tapping. An auditory cue was given to the subjects for the start and stop of the tapping sequences.

#### Data Analysis and Statistics

The fMRI data analysis, performed with SPM8 software, was almost equal as recently described (Christova et al., 2013) and is repeated here in brief. After normalization of the functional data to the mean image spatial smoothing was applied (8 mm full width half maximum Gaussian kernel). For first-level single-subject general linear model (GLM) analysis, a rectangle function of the block onsets with the block duration for each condition (activation, rest) was convolved with a canonical form of the hemodynamic response function. Then all contrast images were analysed in the second level statistics. Using one/two sample *t*-tests the within and between group random effects were assessed. The obtained statistical maps were thresholded at *p* < 0.01, uncorrected, considering only clusters that showed significance at *p* < 0.05, corrected.

Two regions of interest (ROIs) were determined: primary motor (M1) and primary somatosensory cortex (S1). They were based on the differences in the maximum activation areas between the stimulated and the sham group after rPMS. The coordinates were labelled according to Montreal Neurological Institute (MNI) coordinate system. The effect of rPMS on the signal intensity changes was assessed using two factorial ANOVA for each ROI with within subject factor *Time* (PRE, POST1, POST2) and between subject factor *Group* (STIM, NOSTIM). In case of significant interaction or main effect, one factorial ANOVAs for each group with factor Time (PRE, POST1, POST2) were performed and proceeded with by *post hoc* tests.

## Results

Valid data was obtained from all 12 participants of the TMS experiments and from all 30 participants of the fMRI experiments. None of the subjects reported any feeling of pain or discomfort during and following rPMS.

### TMS Results

#### Effect of rPMS on MT

Mean MT values measured at baseline (T0) were 39.6 ± 2.4% for HAND10, 39.8 ± 4.0% for HAND25, and 39.7 ± 3.8% FOOT25. No significant differences were found. Further the ANOVA revealed no effect of the induction protocols on MT measured at *time* levels T1, T2, T3 and T4.

#### Effect of rPMS on MEP Recruitment Curves

**Figure 2:**
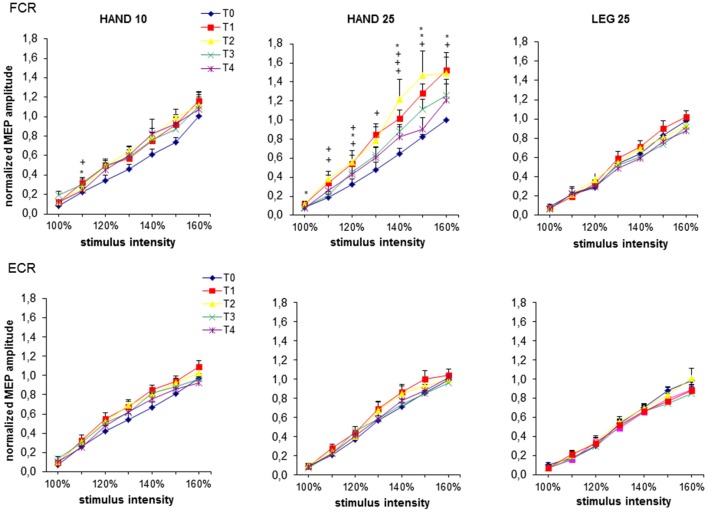
**MEP recruitment curves at baseline (T0), immediately after (T1), 0.5 h (T2), 1 h (T3) and 2 h (T4) after 20 min rPMS.** Upper plots represent the results from the flexor carpi radialis (FCR), lower plots represent the results from the extensor carpi radialis (ECR). MEPs are normalized to the maximum mean MEP at T0 from each subject individually and then pooled for all subjects for each rPMS separately. For each stimulus intensity mean and standard error of mean (SEM) of the normalized MEP amplitude is plotted. The significant differences between T0 and the corresponding post stimulation assessment are presented (*p < 0.05; ^+^*p* < 0.01).

MEP RCs are presented on Figure 2. No significant differences between the three induction protocols were found at *time* level T0. For all stimulation intensities (100–160%) the two-factorial ANOVA at FCR revealed a significant interaction effect of *rPMS* × *time* (*p*s < 0.04). No significant effect was found for ECR for any of the rPMS protocols. Therefore, in addition two-factorial ANOVAs were carried out only for FCR for each protocol with factor *time*. Here protocols HAND25 and HAND10 showed significant effects. For HAND25, significant main effect of *time* was revealed at all stimulation intensities (*p*s < 0.04). This effect was most prominent at time level T2 and slowly returned to the base level at T3 and T4. For HAND10 the ANOVA revealed significant main effect of *time* only from 100 to 120%. Significant main effect of *time* was revealed for stimulation intensities from 100 to 110% (*p*s < 0.03) at time level T2. Accordingly the RC slopes showed significant change only for FCR where significant main effects of *time* (*F*_(4,44)_ = 2.63, *p* < 0.05) and *rPMS* (*F*_(4,44)_ = 6.94, *p* < 0.001) were revealed. Further, for HAND25 the slope steepness was significantly increased at time level T1 57% (*p* < 0.01) and at T2 66% (*p* < 0.05).

#### Effect of rPMS on SICI and ICF

**Figure 3:**
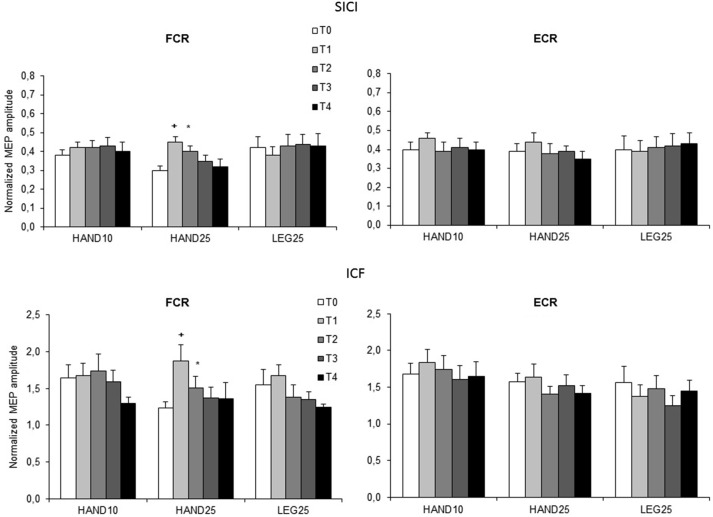
**Normalized paired-pulse responses at baseline (T0), immediately after (T1), 0.5 h (T2), 1 h (T3) and 2 h (T4) after 20 min rPMS.** Upper plots represent the results of both muscles for interstimulus intervals (ISI) of 3 ms (SICI), lower plots represent the results for ISI 13 ms (ICF). Values are normalized for each subject to their corresponding values in single pulse stimulation and plotted as mean (SEM). The significant differences between T0 and the corresponding post stimulation assessment are presented (*p < 0.05; ^+^*p* < 0.01).

The results from paired pulse TMS are shown in Figure 3. No significant differences between the induction protocols were revealed at the *time* level (T0), both for SICI and ICF. Separate ANOVAs were conducted for each muscle and for ISI (3, 13 ms) to reveal the effect of rPMS on SICI and ICF, respectively. For SICI registered from FCR significant interaction effect *rPMS* × *time* was found (*F*_(8,88)_ = 1.68, *p* < 0.05). For ICF significant main effect of *time* (*F*_(4,44)_ = 4.75, *p* < 0.005) was found. For SICI registered from ECR ANOVA showed no significant effects. Follow-up ANOVAs were conducted for FCR separately for each rPMS protocol (HAND10, HAND25 and LEG25). Here a significant main effect of *time* was revealed only for HAND25, both for SICI (*p* < 0.005) and for ICF (*p* < 0.001). The SICI value was increased 50% at T1 and 30% at T2, equivalent to a reduced effect of intracortical inhibition. ICF was increased 52% at T1 and 23% at T2. At T3 and T4 both SICI and ICF returned to baseline levels. HAND10 and LEG25 did not cause any significant changes in SICI and ICF.

### fMRI Results

**Figure 4:**
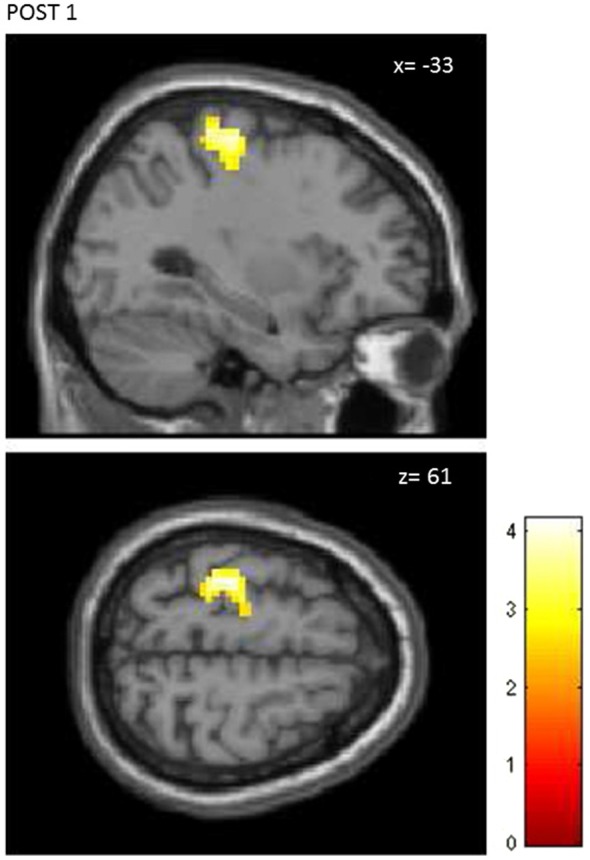
**Random effects between-groups analysis.** STIM vs. NOSTIM group after 25 Hz rPMS at lime level POST1. Significant changes in activation pattern within the left precentral/postcentral gyrus (*p* = 0.01, uncorrected, *p* < 0.05, corrected on cluster level).

The first level analysis showed significant activation-related BOLD response induced by finger tapping for all participants (Ts peak > 12.81), therefore data from all 30 subjects (15 STIM, 15 NOSTIM) were taken for the second level statistical analysis. For both groups across all fMRI assessments, finger tapping activated the left precentral area (M1), left and right postcentral areas (S1), left supplementory motor area (SMA) and left and right cerebellum. The most remarkable differences in the maximum brain activation areas between the stimulated and the sham group after rPMS occurred within the contralateral M1 and S1. At baseline condition (PRE) the activation within these areas was comparable for both groups and in the ROI analysis between-groups differences were not found for M1 (*p* = 0.27) and for S1 (*p* = 0.53). Further random effects analysis showed significant difference in brain activation between the STIM and the NOSTIM group after rPMS (POST1), see Figure 4. Compared to the NOSTIM group an augmented BOLD response within the left sensorimotor cortex (precentral, postcentral) was revealed (*T* = 2.47), cluster peak (MNI, *x* = −33 mm *y* = −25 mm *z* = 61 mm). At the second post stimulation assessment (POST2) this effect was not present any more.

Further, ROI analysis was carried out separately for M1 and S1. Post-stimulation effects were revealed only for M1. ANOVA showed significant interaction effect of *Time × Group* (*F*_(2,56)_ = 3.45; *p* = 0.04). Main effect of *Time* only was found for the STIM group. Significant change in the contrast response between PRE and POST1 (*p* < 0.001) was shown in the *post hoc* analysis. Again at POST2 this effect was not present anymore.

## Discussion

The aim of this study was to advance our knowledge on rPMS-induced neuromodulatory effects within the sensorimotor networks. From the TMS assessments an increase in corticomotor excitability was found after rPMS with a rate of 25 Hz, and in the subsequent fMRI assessment short lasting focal activations within the sensorimotor cortex were detected.

A main finding from the TMS assessments can be ascribed to the induction protocol: there was a facilitation of corticospinal and intracortical excitabilly after 25 Hz rPMS, while there was almost no change in excitability after 10 Hz rPMS. Basically three mechanisms have to be considered for this effect. The first mechanism refers to resonance-like phenomena in the somatosensory cortex as determined with steady-state somatosensory evoked potentials (SSSEP). With mechanical stimulation maximal SSSEP responses were found with stimulus repetition rates at 26 Hz (Snyder, 1992), at 21 Hz (Tobimatsu et al., 1999), and at 27 Hz (Müller et al., 2001). With optical intrinsic signal imaging it was further shown in the animal model that increasing the amplitude of a 25 Hz stimulus led to a proportional increase in the absorbance within the forearm representational area of S1 (Simons et al., 2005). Such results provide further evidence that a 25 Hz induction protocol is more effective to entrain neurons in S1 and in synaptically linked motor areas (Romo et al., 2002), compared to a 10 Hz protocol.

A second mechanism refers to the effect of stimulation frequency in the induction of synaptic plasticity. In neocortical slice preparations a higher frequency (100 Hz) induced LTP, whereas a lower frequency (2 Hz) induced long term depression (LTD; Hess and Donoghue, 1996). In humans analogous cortical neuroplastic effects have been demonstrated with a tactile discrimination task (Ragert et al., 2008): high frequency stimulation (20 Hz) of index finger increased tactile acuity, while low frequency stimulation (1 Hz) decreased acuity. Further in a study where TES was paired with TMS (Pitcher et al., 2003), neuromodulatory post-effects up to 40 min appeared following 30 Hz TES, while there was no effect following 3 Hz TES. Correspondingly our 25 Hz induction protocol should be more effective to evoke LTP-like plasticity within the sensorimotor cortex compared to the 10 Hz protocol.

As a third mechanism, dose dependency may account for this finding. Accordingly a total number of 15,000 single pulses (25 Hz rPMS) was strong enough in order elicit neuromodulatory changes, while this was not the case after the smaller dose of 6000 pulses (10 Hz rPMS). On the other side, the number of pulse trains was equal for both induction protocols. Additional investigations are needed in order to discriminate between factors pulse frequency, number of pulse trains and total number of pulses. In summary, from the current results it cannot be concluded which one of the above three mechanisms is the dominant one. Nevertheless the factum that the 25 Hz induction protocol is much more effective in driving cortical plasticity is well in line with our previous study on mechanical stimulation (Christova et al., 2011).

Another finding from the TMS assessments is the focality of the rPMS-induced effects. Lasting facilitation was found from the MEPs recorded over the target muscle but not from the MEPs over the antagonist. Basically such a result was expected as we used a figure of eight coil to ensure focal stimulation during the rPMS treatments, and accordingly we could observe the pulse train induced wrist and finger flexions. The fMRI assessments also revealed focal post-effects. Interestingly no significant post-effects outside of the sensorimotor area were detected with fMRI, although both sides of the cerebellum became activated during the test motor task. This result shows that a period of afferent input from nerve stimulation, combined with proprioceptive input from finger and wrist movements, was not able to drive post-effects in cerebellar structures. Nevertheless cerebellar structures have been shown to be involved in the processing afferent input (Wardman et al., 2014), and further have been shown to mediate motor cortical plasticity via cerebellothalamocortical pathways (Manto et al., 2006).

Compared to electrical induction protocols, the post-effects induced by rPMS are shorter lasting. After 10 Hz rPMS only the RC at time level T1 displayed some minor post-effect, however this effect cannot be linked exclusively to the cortical level as peripheral excitability was not tested in this study. After 25 Hz rPMS the RCs displayed increased corticospinal excitability up to 60 min, combined with increased intracortical excitability up to 30 min. In comparable TES studies (stimulation at motor level) longer lasting effects on excitability have been described. Charlton et al. (2003) found increased corticospinal excitability 2 h after cessation of 10 Hz TES with a period of 120 min. Golaszewski et al. (2012) demonstrated increased corticospinal and increased intracortical excitability 1 h after cessation of 30 min of whole hand TES. Concerning the time period of stimulation, it appears that longer duration stimulation could result in longer duration post-effects. However in recent experiments with 30 Hz TES (Andrews et al., 2013) it was shown that a shorter period (20 min) was more effective to drive cortical plasticity. Anyway, TES appears to be more attractive than rPMS, at least as it requires less bulky equipment.

Also compared to mechanical induction protocols the post-effects induced by rPMS seem to be less lasting. In the study of Christova et al. (2011) increased corticospinal excitability persisted within 2 h, and increased intracortical excitability persisted within 1 h after 20 min of whole hand vibration stimulation with a frequency of 25 Hz. On the other side, in the study of Steyvers et al. (2003) increased corticospinal excitability persisted just 1 h after 30 min of tendon vibration with a frequency of 80 Hz. Shorter lasting effects were also revealed with fMRI. In the current study an increased BOLD response persisted for some minutes after the treatment, while in a comparable study (20 min vibration stimulation at 25 Hz) an increased BOLD response persisted at least for one hour (Christova et al., 2013).

Thus from the current findings it is concluded that a period of 25 Hz rPMS is well able to drive motorcortical excitability, however for the induction of prolonged neuroplastic effects the current rPMS protocols showed less suited. As the rPMS pulse trains lead to contractions and limb movements without descending corollary discharges in cerebellum (there is no efference copy), inhibition via cerebellar pathways may account for the weaker induction effects. Further studies including theta burst stimulation protocols in order to modulate cerebellar function (Popa et al., 2013) could shed some new light on the subcortical interactions going along with the induction of motor cortical plasticity.

## Conflict of Interest Statement

The authors declare that the research was conducted in the absence of any commercial or financial relationships that could be construed as a potential conflict of interest.

## Footnotes

^1^www.magandmore.com/
